# Navigating the complexities of morality and culture: a critical commentary on the special topics issue “culture and morality: the things we value”

**DOI:** 10.3389/fpsyg.2025.1530299

**Published:** 2025-05-12

**Authors:** Tian Xie, Michael Shengtao Wu

**Affiliations:** ^1^School of Journalism and Communication, Research Center for Intercultural Communication, Wuhan University, Wuhan, China; ^2^School of Sociology and Anthropology, Xiamen University, Xiamen, China

**Keywords:** culture, morality, dynamics, methodological diversity, globalization

This commentary delves into the intricacies of morality within cultural contexts, as explored in the special topics issue “Culture and Morality: The Things We Value” (Wu et al., 2025). It critiques the deterministic perspective of culture on morality, advocating for a more dynamic understanding that incorporates personal agency and power dynamics. Next, it makes a summary and categorizes the 12 articles, published in this special issue, into two themes (e.g., cultural influence on moral behavior, and cultural differences in moral judgments). It also suggests the implications for our understanding of morality within cultural contexts and concludes by underscoring the need for future research to address identified gaps and biases, particularly regarding cultural representation, methodological diversity, intersectionality, power dynamics, and the impact of globalization on moral values. Lastly, it questions the current research agenda and suggests areas for further exploration, such as moral ecology, moral education, methodological rigorousness, and moral identifications in globalization. In conclusion, while the special issue provides valuable insights into the field of culture and morality, it also deserves a more nuanced and critical examination of the interplay between morality and culture.

## Cultural determinism and beyond

The editorial sets the stage for the special issue by outlining the importance of studying morality as a form of social norms that guides human behavior. It emphasizes the variation in moral standards across cultures and the rewards and punishments associated with moral actions. The editorial's contribution lies in its recognition of the importance of cultural context in understanding morality. This is an insightful framework for considering how moral values are shaped and how they influence behavior. By highlighting the role of cultural expectations in moral actions, the editorial opens up a dialogue on the complex relationship between culture and morality.

However, there are also some challenges to the editorial's assumptions and omissions.

Firstly, the universality of moral values vs. cultural relativism. The editorial seems to suggest universality in the importance of morality, yet it fails to sufficiently address the debate between universal moral values and cultural relativism. This commentary argues for a more nuanced understanding that acknowledges the existence of both universal and culturally specific moral values. The special issue could have benefited from an exploration of how universal values, such as human rights, interact with culturally specific values.

Secondly, the role of power dynamics. The editorial does not adequately consider the role of power dynamics in shaping moral narratives. Power structures within and across cultures can significantly influence what is considered moral. For example, dominant groups may impose their moral values on marginalized groups, leading to an unequal distribution of moral authority. This commentary calls for a more critical examination of power in the construction of moral values.

Thirdly, the impact of globalization. Globalization's impact on moral standards is another area where the editorial falls short. As cultures interact and influence each other on a global scale, the special issue could have benefited from an exploration of how these interactions affect moral values. Globalization can lead to the diffusion of moral values, creating new forms of cultural hybridity and moral complexity.

All in all, it seems that the editorial's approach to morality and culture is somewhat deterministic, suggesting a direct correlation between cultural expectations and moral behavior. It overlooks the dynamic and negotiated nature of moral values within cultural contexts. Maybe more effort could be put into accounting for the agency of individuals in interpreting and acting upon moral values, which can vary significantly even within the same cultural group.

## Summary of the 12 articles

The 12 articles in the special issue can be succinctly categorized into two overarching themes: the influence of culture on moral behavior and wellbeing, and the role of cultural differences in shaping moral judgments and social interactions.

The first set of articles provides a comprehensive look at how cultural factors influence moral behavior and wellbeing. Zhou et al. (2023) offer valuable insights into the Chinese context, highlighting the role of collectivism and red culture in shaping subjective wellbeing. Tanaka et al. (2024) extend this discussion by comparing the motivations behind social support provision between European Americans and Japanese individuals, revealing the nuanced ways in which cultural values shape our responses to others' needs. Wu et al. (2023) contribute to this set by examining the detrimental effects of stereotype threat on the motivation of generationally poor individuals to escape poverty, underscoring the real-world implications of psychological research on morality and social mobility. Zhang et al. (2023) delve into the realm of affective forecasting, exploring how subjective socioeconomic status moderates the influence of basic psychological needs satisfaction on the accuracy of predictions about future feelings. Eriksson et al. (2023) provide a historical perspective by examining changes in the appropriateness ratings of everyday behaviors over the past 50 years in the United States, shedding light on the dynamic nature of social norms and their underlying values. Lastly, Taku and Arai (2023) explore the complex interplay between value importance, value congruence, and mental health outcomes, particularly passive suicide ideation, during the COVID-19 pandemic, demonstrating the protective role of certain values in times of crisis.

The second set of articles focuses on the role of cultural differences in shaping moral judgments and social interactions. Chen-Xia et al. (2023) provide a comparative analysis of how individuals from different cultural backgrounds perceive and react to social norm transgressions, highlighting the influence of individualism and collectivism on moral judgments. Zhu et al. (2023) contribute to our understanding of unethical behavior by examining how group-based competition can influence it, with a particular focus on the role of collective efficacy in curbing such behavior. Lin et al. (2024) offer a fascinating exploration of how envy and belief in a just world can mediate and moderate the punishment recommendations for high-status vs. low-status wrongdoers, respectively. Durham et al. (2024) investigate the impact of blame framing and prior knowledge on moral judgments related to historical events, specifically the Tulsa Race Massacre. Their work underscores the importance of context and individual differences in how we process and judge historical moral atrocities. Grigoryev et al. (2024) take a cross-cultural approach to understanding collective action against corruption, revealing both pancultural and culture-specific factors that influence people's willingness to protest government corruption. Finally, Hu et al. (2024) explore the relationship between gratitude and patriotism among college students, with a focus on the mediating role of general life satisfaction and the moderating role of socioeconomic status, adding a dimension of positive psychology to the study of morality.

These articles collectively contribute to a richer understanding of the multifaceted nature of morality within cultural contexts. They highlight the complexity of moral behavior and the importance of considering cultural factors when examining wellbeing, social support, and moral judgments. While the articles provide valuable insights, they also point to the need for further research that can address the gaps and biases identified in the commentary, particularly in terms of cultural representation and methodological diversity.

## Critique and future directions

The special issue has made significant strides in advancing our understanding of morality within cultural contexts, but it also reveals the need for a more inclusive, dynamic, and reflexive approach to this complex field. A critical examination of the articles uncovers several areas that warrant further attention.

One of the most striking observations is the lack of consistency in how the concept of morality is applied across the articles. This inconsistency not only raises questions about the fundamental understanding of morality within the special issue but also echoes this commentary's earlier critiques regarding the need to move beyond potentially simplistic or culturally deterministic perspectives (as suggested in the critique of the editorial's deterministic perspective) and to acknowledge the inherent complexity and variability of moral behavior across different cultural contexts. It highlights the challenges faced when attempting to apply potentially static or culturally-bound (particularly Western) frameworks to capture diverse moral phenomena cross-culturally (e.g., using seemingly universal terms like “justice” or “fairness” based on Western philosophical traditions, without fully accounting for how their specific meanings, implications, and applications can vary substantially depending on local cultural norms, social structures, and historical contexts). Therefore, future research should not seek a single, rigid, universal definition of morality, but rather strive for a more unified approach to defining and measuring it. This approach should accommodate the dynamic and negotiated nature of moral values emphasized earlier in our critique of the editorial's potentially static viewpoint and necessitates integrating diverse perspectives. This might involve developing new theoretical frameworks or adopting a meta-theoretical approach that explicitly incorporates both culturally specific nuances and potential universal dimensions (addressing the universalism vs. relativism debate), thereby helping to move beyond the previously noted reliance on predominantly Western theoretical frameworks.

Regarding the insufficient attention paid to power dynamics and the broader context of globalization, furthermore, there is a noticeable gap across the special issue in considering the moral implications of larger economic systems and political structures. This points to a significant area for future exploration: the 'ecological system of morality,' where moral values and behaviors are understood not only as influenced by cultural norms but also as deeply embedded within, and interacting with, these macro-level societal forces. To develop a more comprehensive understanding of the multifaceted nature of morality, and in response to the call for a more dynamic and contextualized approach highlighted in the critique of the editorial, it is urgent to explore the intersection of morality with these broader societal forces. For example, how do economic inequalities influence moral judgments about wealth distribution? How do political ideologies affect perceptions of fairness and justice? By addressing such questions, researchers can develop a picture of morality that goes beyond the individual or purely cultural level, offering new insights into how power structures and global processes actively shape contemporary moral landscapes.

The special issue's reliance on predominantly Western theoretical frameworks, which, as highlighted by the reviewer and pertinent to our earlier discussion on power dynamics and globalization, is likely intertwined with historical and ongoing global power imbalances, risks overshadowing the diverse moral perspectives present globally. While these established (often Western-derived) theories provide a foundation for understanding morality, their dominance means they may not fully capture the nuances of local cultural phenomena, particularly outside of WEIRD (Western, Educated, Industrialized, Rich, Democratic) contexts. Addressing this limitation requires more than simply applying existing models more broadly; it necessitates a critical examination of how power structures may have shaped the very theoretical lenses we employ. It is essential, as the original text suggests, to recognize potential universal values and principles underlying moral judgments across cultures, alongside the core virtues found in various religions and wisdom traditions worldwide (e.g., the widespread presence of reciprocity principles akin to the Golden Rule in diverse traditions ranging from Confucianism (“己所不欲,勿施于人”) and Hinduism (Mahabharata) to Abrahamic religions and Greek philosophy, alongside broadly shared values concerning justice/fairness, compassion/care, and truthfulness found across numerous ethical systems globally). However, the pursuit of this common moral ground must be conducted through genuinely inclusive global scholarship, rather than assuming universality based on Western-centric perspectives (e.g., Sundararajan, 2020). Therefore, to develop a more nuanced understanding of morality and culture, future research should actively embrace the diversity of moral values and principles across cultures, intentionally incorporating local knowledge and indigenous methodologies not just as novel data points for existing theories, but as valuable sources of theoretical insight in their own right.

Consequently, going beyond the limitations imposed by Western-centric theories, as discussed above, and simultaneously exploring potential universal elements of morality that may transcend cultural boundaries, offers a path toward fostering a more inclusive and accurate representation of global moral diversity. This dualistic approach—attentive to both cultural specificity and potential commonalities, and critically aware of historical power influences on knowledge production— will undoubtedly enrich our understanding of morality. It facilitates a more comprehensive exploration of the dynamic and reciprocal relationship between morality and culture across the globe, and the complexity of the morality-culture interplay was possibly underestimated in the editorial's initial framing. As the original text highlights, this interaction is multifaceted, involving the negotiation of moral meanings within social contexts and the evolution of cultural values through individual and collective moral engagements. Understanding this interplay requires recognizing the agency of individuals—a factor potentially downplayed by deterministic views—and acknowledging the diversity within cultures as people navigate and actively contribute to the moral landscape of their societies.

In terms of practical implications, the articles in the special issue offer valuable insights that could potentially contribute to shaping moral education and informing policy decisions, an aim encouraged by the editorial itself. However, fully realizing this potential requires going beyond the descriptive findings toward a more explicit discussion on how these findings can be effectively applied in these areas. Future research, building upon a more nuanced understanding of the interplay between culture, morality, power dynamics, and globalization as advocated throughout this commentary, should systematically consider the practical implications of its findings. This includes providing concrete recommendations for integrating insights into educational curricula, public policy, and social interventions. For example, taking the findings from the special issue: if gratitude fosters patriotism (see Hu et al., 2024), how can educational programs be designed to cultivate gratitude in culturally sensitive and contextually appropriate ways? If collective action against corruption is influenced by moral obligation (see Grigoryev et al., 2024), what policies—mindful of existing power structures and cultural norms—can be implemented to effectively strengthen this sense of obligation across diverse populations? Addressing the “how” of application necessitates the same sensitivity to context and complexity urged for the research itself.

While the articles in the special issue employ a variety of methodologies, there is still room—and indeed, a pressing need—for greater methodological diversity and rigorousness. This need arises directly from the challenges highlighted earlier, particularly the call to understand morality as a dynamic, context-dependent, and multifaceted phenomenon. Future research should more frequently consider employing:

Mixed-methods approaches: To capture both the breadth and depth of moral experiences, integrating quantitative findings with rich qualitative insights into cultural nuances and subjective meanings—essential for navigating the complexity discussed previously.

Longitudinal studies: To track the dynamic evolution of moral values and behaviors over time within individuals and cultures, moving beyond the static snapshots that cross-sectional studies often provide and addressing the critique of potentially deterministic or overly stable views of culture's influence.

Experimental designs: To establish causal relationships and test specific hypotheses about moral judgment and behavior under controlled conditions, allowing for a more rigorous examination of the factors shaping morality.

Additionally, the strategic use of big data and advanced analytical techniques could offer powerful new insights into the large-scale dynamics of moral behavior and cultural norms, potentially shedding light on the broad impacts of globalization and the functioning of the 'moral ecosystem' at societal levels. Embracing a wider methodological toolkit is crucial for developing a more robust, comprehensive, and globally relevant understanding of moral behavior.

Finally, drawing together the threads of critique concerning power dynamics, the impact of globalization, the limitations of Western-centric frameworks, and the need for a more complex, contextualized understanding, it becomes unequivocally essential to integrate intersectionality, power dynamics, and globalization into the study of morality and culture. Ignoring these intersecting factors, especially the pervasive influence of power which, as noted earlier, is intrinsically linked to the dominance of certain theoretical perspectives, risks producing incomplete or even distorted accounts of moral life. Future research must therefore actively examine how these factors interact to shape moral values, experiences, and behaviors. For example:

How do intersecting social identities such as gender, race, and class co-construct different moral realities and influence judgments about social justice within specific cultural and historical contexts?

How do power imbalances—operating at interpersonal, institutional, and global levels as highlighted in our critique—affect moral decision-making, particularly in intercultural interactions or situations marked by inequality?

By systematically incorporating these broader, interacting factors, researchers can move toward developing the truly nuanced, critically informed, and globally relevant understanding of the complexities of morality that this commentary advocates for—an understanding that acknowledges both the diversity within cultures and the overarching structures that shape our interconnected world.

In conclusion, the special issue has provided a valuable contribution to the field of morality and culture, but it also highlights the need for a more inclusive, dynamic, and reflexive approach to studying morality across cultures. By addressing the challenges and gaps identified in this commentary, future research can build upon the foundation laid by the special issue to develop a more comprehensive understanding of the complexities of morality and culture. This commentary has aimed to provide such a perspective, offering both critique and direction for future research. By considering the implications of the research findings for school and moral education, as encouraged by the editorial, we can work toward fostering a more just and equitable society that values the diversity of moral perspectives and cultural experiences.
